# Fetal Liver Blood Flow Distribution: Role in Human Developmental Strategy to Prioritize Fat Deposition *versus* Brain Development

**DOI:** 10.1371/journal.pone.0041759

**Published:** 2012-08-22

**Authors:** Keith M. Godfrey, Guttorm Haugen, Torvid Kiserud, Hazel M. Inskip, Cyrus Cooper, Nicholas C. W. Harvey, Sarah R. Crozier, Sian M. Robinson, Lucy Davies, the Southampton Women's Survey Study Group, Mark A. Hanson

**Affiliations:** 1 Institute of Developmental Sciences, University of Southampton, University Hospital Southampton NHS Foundation Trust, Southampton, United Kingdom; 2 Medical Research Council Lifecourse Epidemiology Unit, University of Southampton, University Hospital Southampton NHS Foundation Trust, Southampton, United Kingdom; 3 Southampton National Institute for Health Research Biomedical Research Centre, University Hospital Southampton NHS Foundation Trust, Southampton, United Kingdom; 4 Department of Obstetrics, Oslo University Hospital - Rikshospitalet and University of Oslo, Oslo, Norway; 5 Department of Obstetrics and Gynecology, Haukeland University Hospital, Bergen, Norway; 6 Department of Clinical Medicine, University of Bergen, Bergen, Norway; The Ohio State Unversity, United States of America

## Abstract

Among primates, human neonates have the largest brains but also the highest proportion of body fat. If placental nutrient supply is limited, the fetus faces a dilemma: should resources be allocated to brain growth, or to fat deposition for use as a potential postnatal energy reserve? We hypothesised that resolving this dilemma operates at the level of umbilical blood distribution entering the fetal liver. In 381 uncomplicated pregnancies in third trimester, we measured blood flow perfusing the fetal liver, or bypassing it via the ductus venosus to supply the brain and heart using ultrasound techniques. Across the range of fetal growth and independent of the mother's adiposity and parity, greater liver blood flow was associated with greater offspring fat mass measured by dual-energy X-ray absorptiometry, both in the infant at birth (r = 0.43, P<0.001) and at age 4 years (r = 0.16, P = 0.02). In contrast, smaller placentas less able to meet fetal demand for essential nutrients were associated with a brain-sparing flow pattern (r = 0.17, p = 0.02). This flow pattern was also associated with a higher degree of shunting through ductus venosus (P = 0.04). We propose that humans evolved a developmental strategy to prioritize nutrient allocation for prenatal fat deposition when the supply of conditionally essential nutrients requiring hepatic inter-conversion is limited, switching resource allocation to favour the brain if the supply of essential nutrients is limited. Facilitated placental transfer mechanisms for glucose and other nutrients evolved in environments less affluent than those now prevalent in developed populations, and we propose that in circumstances of maternal adiposity and nutrient excess these mechanisms now also lead to prenatal fat deposition. Prenatal developmental influences play important roles in the human propensity to deposit fat.

## Introduction

At birth the human fetus has the highest percentage of body fat [1] but also the largest brain of any primate species [2]. During fetal life, the growth of these organs has to be preserved, but also to be regulated as part of a range of adaptive responses which prepare the offspring for life after birth. Hence, in response to prenatal challenges such as inadequate nutrient supply, ‘strategic decisions’ need to be made regarding prioritization of resources. These include immediate protection of vital organs such as the brain, but must also enable deposition of fat, necessary for postnatal thermoregulation and thought to support continued brain development and metabolism during periods of nutrient restriction after birth [1], [3]. In this study we investigated the role of the perfusion of the fetal liver in such adaptive responses to inadequate nutrient supply associated with smaller placental size, while also taking account of the role of excessive nutrient supply associated with maternal adiposity.

**Figure 1:**
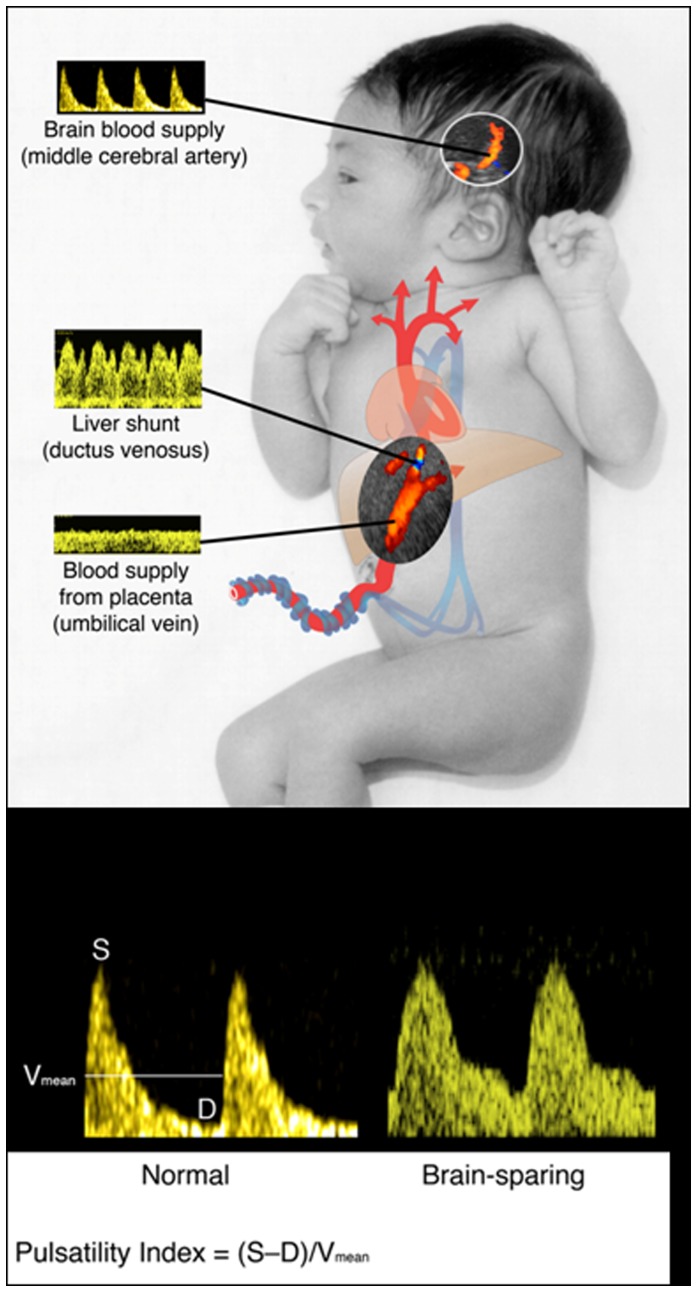
Diagrammatic representation of the fetal circulation, showing blood flow to the fetal liver and brain. (*A*) Nutrient-rich blood returning from the placenta in the umbilical vein either perfuses the fetal liver or bypasses it through the shunt of the ductus venosus. (*B*) “Brain-sparing” is associated with cerebral vasodilation, altering the blood flow velocity waveform in the middle cerebral artery, and lowering the pulsatility index. The figure is modified from Ref 20; reproduced with permission.

The flow of nutrient-rich blood from the placenta divides as it enters the fetus, either to perfuse the fetal liver, or to bypass it via the ductus venosus (Fig. 1). Ductus venosus blood flow is directed preferentially through the foramen ovale to the left atrium, thence to the left ventricle and ascending aorta to supply the coronary and cranial vascular beds [4], [5]. Under circumstances of reduced oxygenation or nutrition, a higher proportion of blood from the umbilical vein bypasses the liver and perfuses the head and neck of the fetus, prioritizing oxygen and nutrient delivery to the brain as part of the so-called ‘brain-sparing’ effect [6], [7]. Such changes in regional blood flow, which reduce liver blood flow and maintain brain and heart metabolism were first described in the diving seal [4]. In fetal sheep, experimental manipulations that reduce liver blood flow diminish hepatic substrate and growth factor synthesis, impairing soft tissue accretion in the body as a whole [8]. In growth-restricted human fetuses, such brain-sparing responses are associated with cerebral vasodilation (measured using Doppler ultrasound as a low pulsatility index in the middle cerebral artery [5]) and with greater ductus venosus shunting (Fig. 1) [6], [7]. Remarkably, it is not known whether such adaptive changes in brain and liver blood flow occur in normally growing fetuses, although there are large variations in the proportion of placental blood perfusing or bypassing the liver in late gestation in such fetuses. Because the fetal liver is critical to fatty acid synthesis *in utero,* we hypothesized that, if adaptive responses affecting blood flow do occur during normal human fetal development, they will affect fat deposition manifest at birth. Furthermore, because it has been suggested that propensity for later obesity may be partly determined by both inadequate and excessive nutrient supply before birth [9], [10], it is possible that variations in fetal adaptive responses might also be reflected in fat deposition in childhood. As the balance between fetal nutrient demands and materno-placental nutrient supply is influenced by fetal gender (greater demand in male fetuses, as they grow faster than female fetuses [11]), and placental size (reduced supply if the placenta is smaller [12], [13]) we have examined fetal adaptive responses in relation to gender and placental weight, and determined relationships with adiposity at birth and at age 4 years.

## Results

In 381 singleton low-risk pregnancies in which the mother's body fatness had been characterized prospectively before conception [14], we measured blood flows from the placenta (umbilical venous flow) and through the ductus venosus in late gestation, followed by neonatal anthropometry and subscapular skinfold thickness. We measured the middle cerebral artery pulsatility index (MCA PI) in 213 of the fetuses.

### Fetal cerebral and ductus venosus blood flows

**Figure 2:**
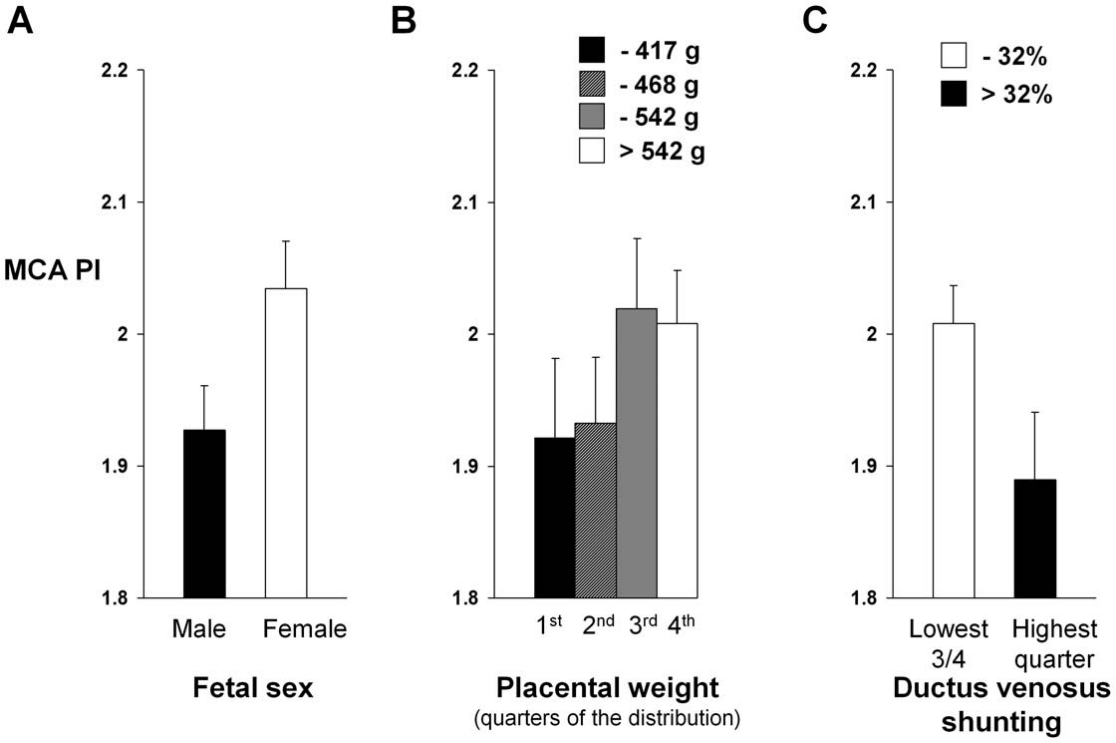
Brain-sparing blood flow pattern in relation to fetal sex, placental weight and liver shunting. Measurements of middle cerebral artery pulsatility index (MCA PI) showed evidence for brain-sparing (low MCA PI) in (*A*) male fetuses (P = 0.03) (Bars represent mean values & SEM, n = 110 and 103, for males and females, respectively), and in (*B*) those with smaller placentas (P = 0.02) (Bars represent mean values & SEM, n = 49, 49, 49, and 48, respectively). (*C*) Greater ductus venosus liver shunting was also related to brain-sparing; MCA PI was lower in fetuses in the highest quarter of the distribution of ductus venosus liver shunting when compared with those in the lowest three-quarters of the distribution (P = 0.04) (Bars represent mean values & SEM, n = 53 and 160, respectively).

There was evidence for brain-sparing in male fetuses and in those with a smaller placental size. Thus MCA PI was lower in male than in female fetuses (P = 0.03) (Fig. 2A), and in those with smaller placenta weights (r = 0.17, P = 0.02) (Fig. 2B and Figure S1). Shunting of blood away from the liver and through the ductus venosus was related to this brain-sparing, with a lower MCA PI in fetuses in the highest quarter of the distribution of ductus venosus shunting (>32%) when compared with those in the lowest three-quarters of the distribution (P = 0.04) (Fig. 2C) but without a significant association across the whole distribution of ductus venosus shunting (r = −0.06, P = 0.4; Figure S2).

### Fetal cerebral and ductus venosus blood flows and neonatal adiposity

Brain-sparing and ductus venosus liver shunting reduce fetal liver blood flow, so next we investigated whether a brain-sparing blood flow distribution was associated with diminished fetal fat deposition. In contrast to previous observations in growth restricted fetuses [15], in this population of low-risk pregnancies lower fetal MCA PI was not related to sex-adjusted birthweight, crown-heel length or head/abdominal circumference ratio; lower fetal MCA PI was, however, associated with lower neonatal subscapular skinfold thickness (r = 0.24, P<0.001)(Figure S3) and fetuses in the highest quarter of the distribution of ductus venosus liver shunting had a thinner neonatal subscapular skinfold thickness than those in the lowest three-quarters of the distribution (4.7 vs 5.1 mm, P = 0.001). Figure S4 suggests that, above a threshold, shunting blood to preserve nutrient delivery to the brain compromises fetal body fat deposition.

### Fetal liver blood flow and neonatal adiposity

**Figure 3:**
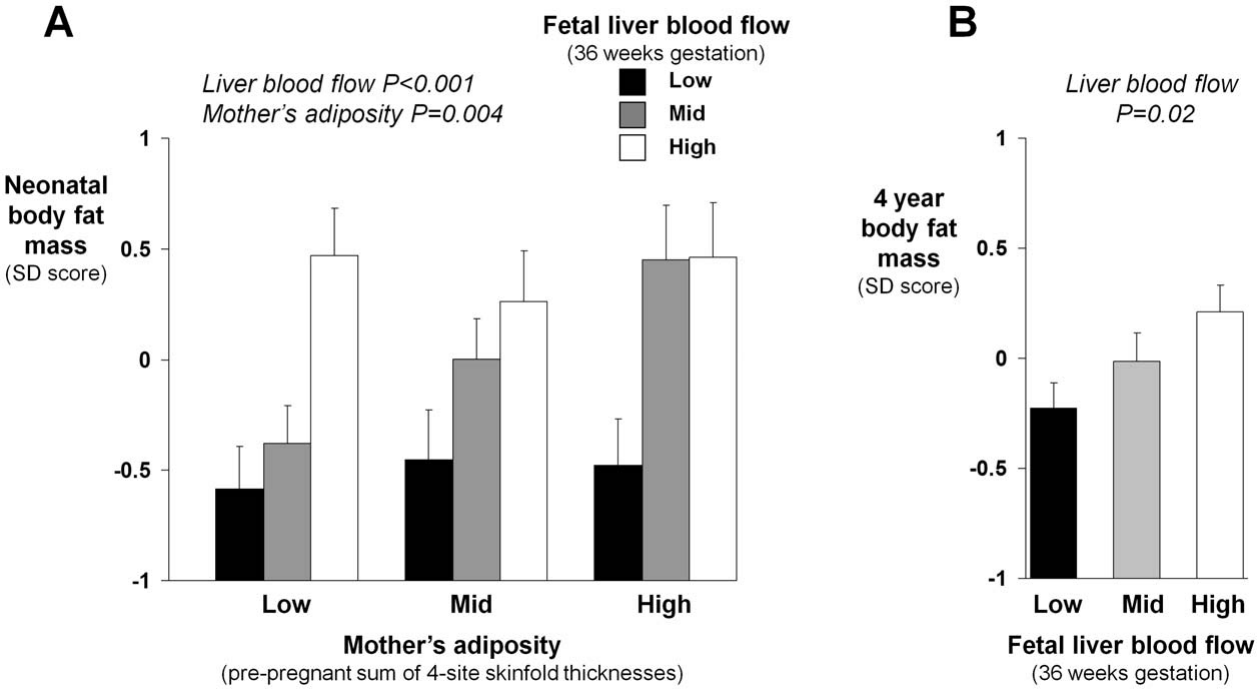
Relations of fetal liver blood flow and mother's adiposity to prenatal fat deposition. (*A*) Greater fetal liver blood flow at 36 weeks gestation was associated with greater neonatal body fat mass at every level of mother's pre-pregnancy adiposity. P values are for a simultaneous analysis in which both greater fetal liver blood flow and greater mother's adiposity had strong associations with greater neonatal body fat mass (Groupings are thirds of the distributions. P values are for analysis of the continuously distributed variables. Bars represent mean values & SEM, n = 15, 15 and 21 for low, 19, 13 and 18 for average, and 17, 23 and 10 for high mother's adiposity groups, respectively). (*B*) The effect of fetal liver blood flow on body fat mass persisted into early childhood, with greater fetal liver blood flow also being associated with body fat mass at age 4 years (Groupings are thirds of the distribution. P value is for analysis of the continuously distributed variable. Bars represent mean values & SEM, n = 61, 67 and 69, respectively).

Umbilical venous blood that is not shunted through the ductus venosus perfuses the fetal liver (Fig. 1). We therefore investigated whether greater fetal liver blood flow was associated with increased fetal fat deposition. We utilized the neonatal subscapular skinfold thickness measurements (n = 378 infants) and estimated total fat mass and % body fat measured by dual-energy X-ray absorptiometry (DXA) (available for 152 infants, and adjusted for the child's sex and gestation at birth [14]). We found strong, graded associations between greater liver blood flow and both higher neonatal subscapular skinfold thickness (r = 0.28, P<0.0001), and greater DXA neonatal total fat mass (r = 0.43, P<0.0001) (Fig. 3A) and % body fat (r = 0.40, P<0.0001). These associations were similar when using fat mass index as a measure of neonatal adiposity (r = 0.41, P<0.0001) and in the full sample and those without MCA PI data (e.g. regression coefficients for fetal liver blood flow vs neonatal subscapular skinfold thickness 0.68 and 0.62 mm/(sqrt)ml/min, respectively). As expected, greater body fatness of the mother (pre-pregnancy four-site skinfold thicknesses) was associated with greater neonatal body fat mass. The association between fetal liver blood flow and neonatal body fat mass was, however, independent of, and stronger than, the association between the mother's body fatness and that of her infant (Fig. 3A). Fetal liver blood flow predicted neonatal body fat mass at every level of mother's adiposity (Fig. 3A). Differences between groups were large: the Z-score of neonatal body fat mass was 0.46 in those with high fetal liver blood flow and high mother's adiposity and −0.58 in those with low liver blood flow and low mother's adiposity. In a simultaneous multiple regression analysis of the continuously distributed data, neonatal body fat mass increased with both higher fetal liver blood flow (P<0.001) and greater mother's pre-pregnancy body fatness (P = 0.004). Taking account of maternal age, parity, reported general health, smoking and social class had little effect on these relations, or on the other associations described above.

### Fetal liver blood flow and adiposity at age 4 years

In 197 of the 381 infants a DXA scan was subsequently performed at a median age of 4.1 years. Adjusting for the child's sex and age, greater fetal liver blood flow was associated with greater total fat mass at age 4 years (r = 0.16, P = 0.02) (Fig. 3B); fetal liver blood flow was more weakly associated with % fat mass (r = 0.12, P = 0.11) and total lean mass (r = 0.10, P = 0.15) at age 4 years. Ductus venosus shunting and the mother's adiposity had no associations with the child's fat mass at age 4 years (P = 0.24 and P = 0.46, respectively), and taking account of the mother's adiposity had little effect on the association between fetal liver blood flow and the child's total fat mass (adjusted r = 0.17, P = 0.02). The above associations were similar when using fat mass index as a measure of adiposity and in the full sample and those without MCA PI data (regression coefficients for fetal liver blood flow vs 4 year DXA fat mass 0.00039 kg/(sqrt)ml/min in both groups).

## Discussion

**Figure 4:**
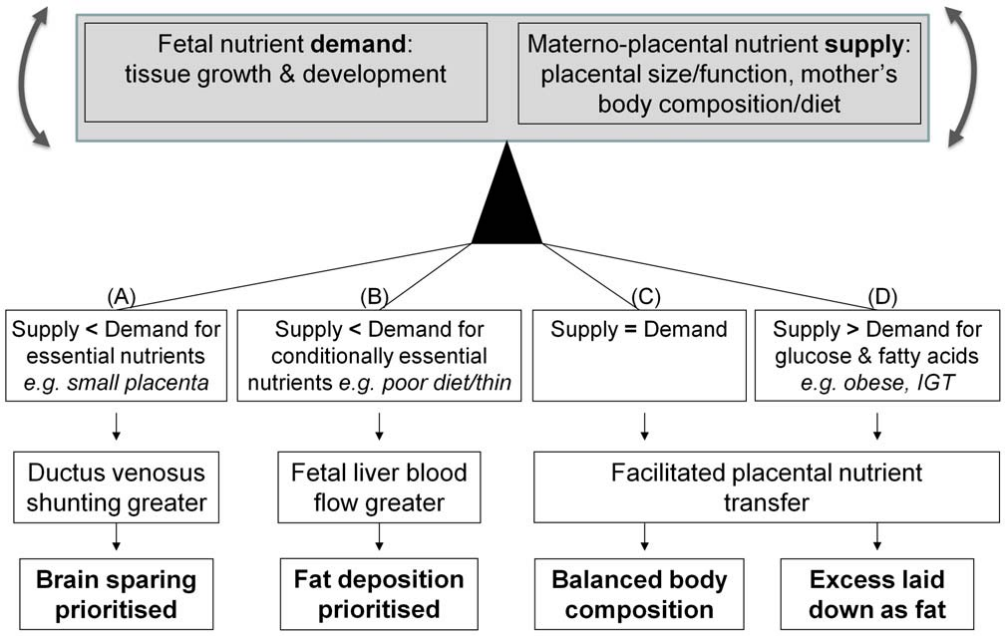
Suggested developmental strategies associated with imbalances between fetal nutrient demand and materno-placental nutrient supply. The distribution of placental blood flow is based on nutritional status, with ductus venosus liver shunting and brain-sparing if the fetal demand for essential nutrients exceeds placental supply (A). However, when supply of conditionally essential nutrients is inadequate, the strategy is to prioritize liver blood flow, enabling hepatic nutrient interconversions and the synthesis of fatty acids required for fat deposition; in this circumstance fat deposition is prioritized as it is needed for neonatal thermoregulation and as a buffer for brain development during subsequent periods of limited nutrient supply (B). In environments less affluent than those now prevalent in developed populations, facilitated placental transfer mechanisms evolved for glucose and other nutrients to enable materno-placental nutrient supply to meet fetal nutrient demand, resulting in optimal fetal body composition (C); however, in circumstances of nutrient excess (such as maternal adiposity and impaired glucose tolerance (IGT)) these mechanisms also lead to prenatal fat deposition (D).

We previously showed that distribution of nutrient-rich placental blood between liver and the ductus venosus liver shunt, controlled by haemodynamic [16], [17] and neuro-hormonal mechanisms [18], [19] is affected by maternal thinness [20], which itself is linked to dyslipidemia, impaired glucose tolerance and susceptibility to metabolic disease in later life in the offspring [21], [22]. Table 1 summarises the principal findings of the present study. Variations in the distribution of nutrient-rich placental blood associated with placental size and fetal adiposity lead us to speculate about a possible mechanistic framework integrating our observations (Fig. 4). In this we hypothesise that the balance between fetal nutrient demand and materno-placental nutrient supply may alter the distribution of umbilical venous blood flow, with implications for fetal body composition. Umbilical venous blood flow distribution is related to nutritional status, with so-called ‘brain-sparing’ mechanisms [4] coming into play only if placental supply does not meet fetal demand for essential nutrients that cannot be synthesised by the fetal liver, such as oxygen [4]. This tends to occur when the fetal demand is relatively high (e.g. male fetus, Fig. 2A) and the supply capacity and umbilical venous blood flow is constrained (e.g. small placenta, Fig. 2B). However, when the mother has a poor diet or is thin it is the supply of conditionally essential nutrients that is limiting and the strategy is to prioritize liver blood flow in these circumstances (as reported previously) [20], enabling hepatic nutrient interconversions and the synthesis of the fatty acids required for fat deposition. Conditionally essential nutrients are those essential for metabolism and growth, but which can be generated by hepatic conversion of other nutrients if the dietary supply is inadequate [23], [24]; examples include amino acids such as glycine and long chain fatty acids such as docosahexaenoic acid. Fetal liver blood and fat deposition (Fig. 3) is prioritized *in utero* as adipose tissue is needed for neonatal thermoregulation and as a buffer for brain development if there are subsequent periods of limited nutrient supply. In keeping with this model, we have recently shown that increased fetal liver blood flow is associated with fetal macrosomia in the third trimester [25]. Our model (Fig. 4) further hypothesises that, in environments less affluent than those now prevalent in developed populations, facilitated placental transfer mechanisms have evolved for glucose and other nutrients to enable materno-placental nutrient supply to meet fetal nutrient demand, resulting in optimal fetal body composition. However, we propose that in circumstances of nutrient excess these mechanisms also lead to prenatal fat deposition, reflected in the association we found between maternal adiposity and neonatal fat mass (Fig. 3A).

**Table 1:** Principal findings of the study.

Exposure	Outcome
Male gender and smaller placental size	Lower middle cerebral artery pulsatility index
Lower middle cerebral artery pulsatility index	Lower neonatal subscapular skinfold thickness
High ductus venosus shunting	Lower middle cerebral artery pulsatility index; lower neonatal subscapular skinfold thickness
Greater fetal liver blood flow	Higher neonatal subscapular skinfold thickness; greater DXA total fat mass both at birth and at age 4 years

We recruited children from a free-living population cohort and used objective measures of postnatal adiposity. However, there are several limitations to our study. Intrauterine ultrasound measurements are prone to a certain amount of error, but the Doppler blood flow measurements were made by a single experienced operator (GH) following internationally agreed guidelines and repeatability was good. Secondly, we were only able to follow up a proportion of the original group at age 4 years, but the children who underwent the 4 year assessment did not differ in terms of their fetal liver blood flow or adiposity at birth from those who did not. Moreover, as the analysis is based on internal comparisons it is difficult to envisage how this would have spuriously shown an association between fetal liver blood and postnatal adiposity. Thirdly, a range of potential confounders were considered but residual confounding cannot be excluded. Fourthly, we used DXA to measure fat mass. This technique is associated with technical limitations in children and is hampered by their tendency to move. However, we used specific paediatric software, and movement artefact was modest and uniform across the cohort; those few children with excessive movement were excluded from the analysis. Fifthly, the association between high ductus venosus liver shunting and thinner neonatal subscapular skinfold thickness was only seen above a threshold and the possibility of an artefactual finding must be born in mind. The clear, graded association between fetal liver blood flow and adiposity was not seen for ductus venosus shunting; we speculate that this may be the result of a direct link between hepatic perfusion and adipose tissue deposition as opposed to increased ductus venosus shunting only occurring above a threshold of constrained fetal nutrient supply.

Our proposal that alterations in fetal blood flow distribution may have wider implications is supported by experimental studies in animals showing that hepatocyte gene expression can be altered by changes in fetal liver blood flow [5] and by maternal diets with a low protein content [26]. Maternal low protein diets have also been shown to alter epigenetic processes in hepatocytes in the offspring [27], with permanent consequences including altered liver carbohydrate and fat metabolism [27] and a predisposition to adult obesity and reduced lifespan [26], [28]. Moreover, a reduction in liver glucocorticoid receptor and fibrinogen gene expression caused by a low protein maternal diet was confined to the left hepatic lobe [29]; in circumstances of high ductus venosus liver shunting there is differential perfusion of the right and left hepatic lobes, with preferential perfusion of the left hepatic lobe by umbilical blood [30].

Adiposity in childhood is associated with the risk of adult obesity [31]. The association of fetal liver blood flow with fat mass not only in the newborn but also at four years of age in this cohort with birth weights within the normal range is thus of concern in relation to obesity and the risk of associated diseases later in life.

Our results lead us to propose that in the normal human fetus a developmental strategy of allocating umbilical venous blood flow to the liver, to overcome an inadequate supply of conditionally essential nutrients and prioritize fat deposition, brings with it important metabolic consequences which can have lasting effects on body composition. In the human infant, the demands of a big brain have not only led during evolution to the development of fetal responses which preserve nutrient delivery to the brain when the materno-placental nutrient supply of essential nutrients cannot meet fetal nutrient demand, but also to a need to deposit fat in order to buffer brain development during periods of nutrient restriction in postnatal life. We hypothesise that evolution of this strategy has brought with it a predisposition to obesity and later diabetes in contemporary societies with abundant nutrition in later postnatal life [32]. Because the strategy is a fundamental aspect of human biology, it occurs to a degree across the range of infant growth and development, and could be an important determinant of the risk of obesity at the population level. Furthermore the strategy is compounded by the evolution of facilitated placental transfer mechanisms for glucose and other nutrients which now also lead to fetal adipose tissue deposition in the circumstance of nutrient excess associated with maternal adiposity now prevalent in developed communities. Although our hypothesis is supported by experimental data from animal studies, direct causality cannot be inferred from observational data. Nonetheless the findings add to an increasing evidence base suggesting that particular developmental influences acting before birth play important roles in the human propensity to deposit fat; understanding this fundamental biology in early life may be valuable in the prevention of obesity and early detection of those at particular risk.

## Materials and Methods

### Ethics Statement

All aspects of the study were approved by the Southampton and SW Hants Local Research Ethics Committee, the women gave written informed consent, and the work was performed according to the Declaration of Helsinki.

### Methods

We studied singleton uncomplicated pregnancies in women whose body composition (four-site skinfold thicknesses) and characteristics (including parity, smoking and social class) had been measured pre-pregnancy in the population-based Southampton Women's Survey [14]. In 410 pregnant women, we used ultrasound Doppler to measure blood flows in the umbilical vein (UV) and ductus venosus (DV) [20], [33], [34]. We obtained complete data in 381 subjects (93%) and derived % shunting (ratio DV flow/UV flow) and liver blood flow (UV flow – DV flow). In 213 fetuses, fetal pulsatility index (PI) was measured in the middle cerebral artery [35]. We used a computerised algorithm to derive gestational age using menstrual data (66%) or, when these were uncertain or discrepant with ultrasound assessments, fetal anthropometry in early pregnancy. Median (10^th^–90^th^ centile) gestation at cerebral and venous blood flow measurement were 34^+4^ weeks (33^+6^–35^+1^) and 36^+1^ weeks (35^+1^–37^+3^), respectively; median liver blood flow, DV shunting and pulsatility index in middle cerebral artery were 150.9 (87.3–239.4) ml/min, 24.1 (12.0–42.0) % and 1.98 (1.52–2.45), respectively; median maternal age, pre-pregnant BMI and sum of skinfold thicknesses and infant birthweight were 30 years (25–35), 23.8 kg/m^2^ (21.9–26.5), 62.4 mm (47.6–86.5) and 3485 g (2875–4160), respectively. 50% were primparous, 26% were smokers, 98% reported being in very good, good or fair health and 13% were of lower socioeconomic status (Registrar General Social Class IV/V [36]).

### Doppler measurements

We measured internal vessel diameter (late-diastole) and time-averaged maximum velocity (TAMX) (insonation angle <30°) in the intra-abdominal UV (straight portion, before hepatic parenchymal branches) and at the DV inlet (Acuson Sequoia, CA). UV TAMX was obtained during a 3–5 sec period or, if flow was pulsatile, as the mean during three heart cycles. DV TAMX was calculated as the mean during three heart cycles. Blood flow (Q) was calculated as Q = h·(D/2)^2^·π·TAMX, where D = vessel diameter (mean of 5–10 measurements [37]) and h = spatial blood velocity profile coefficient (UV = 0.5; DV = 0.7) [30], [38]–[40]. Intra-class correlation coefficients (random-effects regression) to assess intra-observer variation were 0.97 and 0.96 for the UV and DV diameter, respectively.

Pulsatility index was measured in the proximal segment of the middle cerebral artery; during fetal quiescence, we calculated the mean during three consecutive heart cycles, keeping the insonation angle as close to 0° as possible [35].

### Neonatal anthropometry and dual-energy x-ray absorptiometry

Infant weight and trimmed placental weight [41] were measured at birth using electronic scales, and neonatal subscapular skinfold thickness was measured within 48 hours of delivery using a skinfold calliper (Holtain) [14]. Measurements were repeated three times and the mean used in analyses. Regular inter-observer variation studies were carried out during the fieldwork. Subjects registered with a stratified sample of primary care practitioners were invited to have their infants' body composition measured by dual-energy x-ray absorptiometry within 20 days of birth (Lunar DPX-L (GE Corporation, Wisconsin); effective radiation dose 0.31 microsieverts). Total and proportionate fat mass were derived from a whole-body scan, using pediatric software (version 4.7c), and fat mass index derived as fat mass/height^2^. 152 infants (87 with MCA PI data) were scanned while swaddled in a towel to help reduce movement artifact. 197 of the 381 infants (106 with MCA PI data) had a further DXA measurement of fat mass at age 4 years (Hologic Discovery, Hologic Inc., Bedford, MA, USA; effective radiation dose 26.7 microsieverts); to help reduce movement artifact, the children were shown an age-appropriate suitable DVD cartoon. The instruments were calibrated daily; short- and long-term coefficients of variation were 0.8% and 1.4%, respectively. Fetal and neonatal characteristics were similar for subjects followed up and not followed up at age 4 years (e.g. median fetal liver blood flow 151.5 vs 148.2 ml/min and mean neonatal subscapular skinfold thickness 4.97 vs 4.96 mm, respectively).

### Statistics

Statistical techniques used in analyses were t-tests for analyses of dichotomous outcomes, and Pearson's correlation (r) and multivariate regression for analyses of continuous outcomes. Where appropriate, variables were transformed using logarithms or square roots to satisfy statistical assumptions of normality. We used linear regression to adjust the neonatal DXA measurements of fat mass for sex and gestation at birth, and to adjust the 4-year fat mass measurements for sex and age at the measurement. For ease of interpretation, transformed DXA fat mass values were standardised internally and the results shown as standard deviation (SD) scores. Except where stated, statistical analyses are of the continuously distributed data; figures show grouped data to illustrate effect sizes and whether or not associations are graded across the distribution. Analyses were performed using Stata 10.0SE (Stata Corp, Texas). We first used the MCA PI measurements to examine for evidence of brain sparing in relation to fetal gender, placental size and altered liver blood flow, and examined the relations of MCA PI and ductus venosus shunting with neonatal anthropometry and body composition; as umbilical venous blood that is not shunted through the ductus venosus perfuses the fetal liver we next related fetal liver blood flow to the offspring's body composition at birth and at age 4 years, taking account of the mother's adiposity and other potential confounding influences.

## Supporting Information

Figure S1 **Scatterplot of fetal middle cerebral artery pulsatility index (MCA PI) in late gestation in relation to placental weight.**
(TIF)

Figure S2 **Scatterplot of fetal middle cerebral artery pulsatility index (PI) in late gestation in relation to (square root) ductus venosus shunting.**
(TIF)

Figure S3 **Scatterplot of neonatal subscapular skinfold thickness in relation to fetal middle cerebral artery pulsatility index (PI) in late gestation.**
(TIF)

Figure S4 **Neonatal subscapular skinfold thickness according to fourths of the distribution of ductus venosus shunting at 36 weeks gestation.** Values are means and SEM.
(TIF)
