# Supplementary figures and images for: Fetal Liver Blood Flow Distribution: Role in Human Developmental Strategy to Prioritize Fat Deposition versus Brain Development
Source: PLoS One. 2012 Aug 22;7(8):e41759. doi: 10.1371/journal.pone.0041759 (PMC3425554; doi:10.1371/journal.pone.0041759)

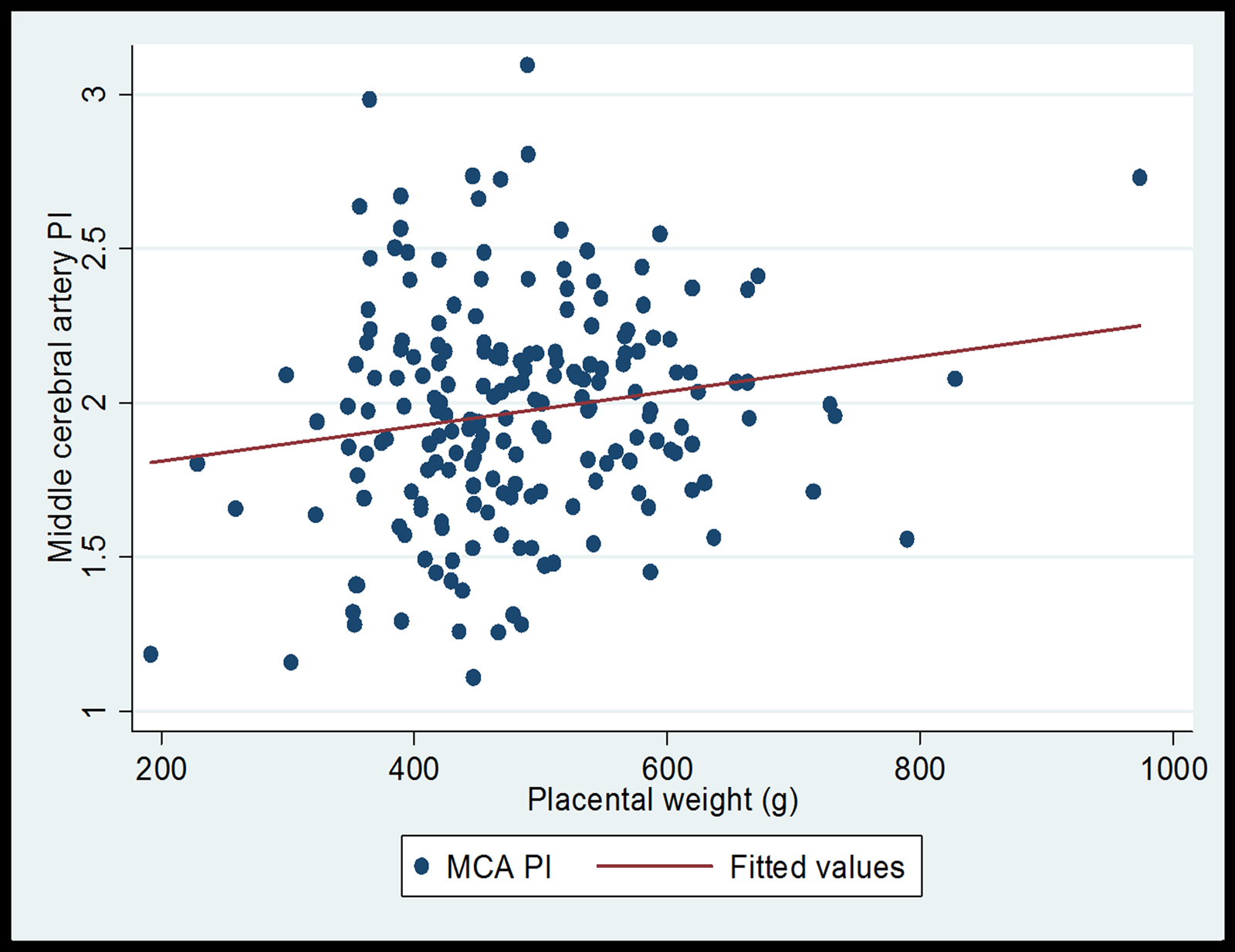

Supplement: Figure S1 — Scatterplot of fetal middle cerebral artery pulsatility index (MCA PI) in late gestation in relation to placental weight. (TIF) [file pone.0041759.s001.tif]

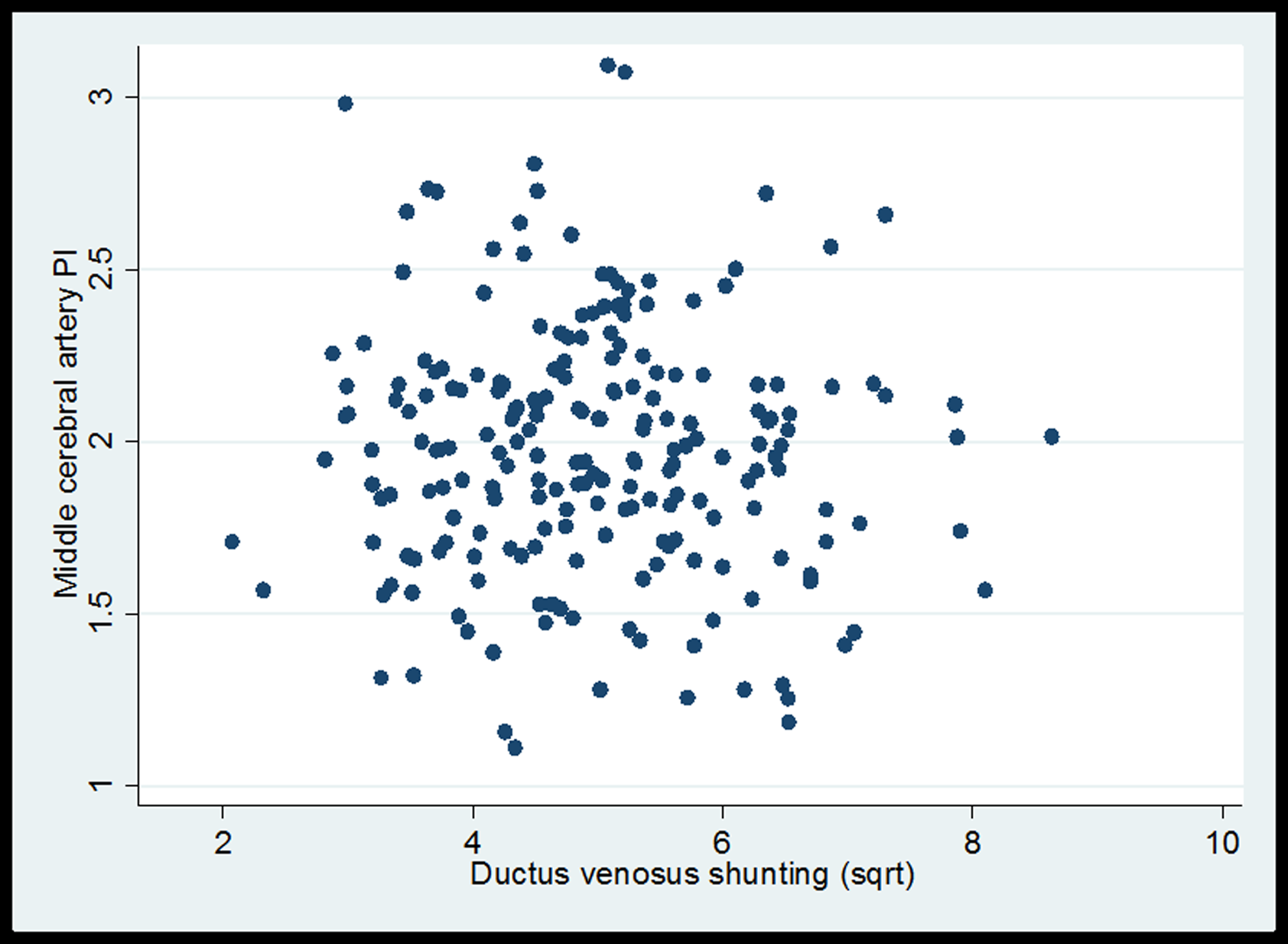

Supplement: Figure S2 — Scatterplot of fetal middle cerebral artery pulsatility index (PI) in late gestation in relation to (square root) ductus venosus shunting. (TIF) [file pone.0041759.s002.tif]

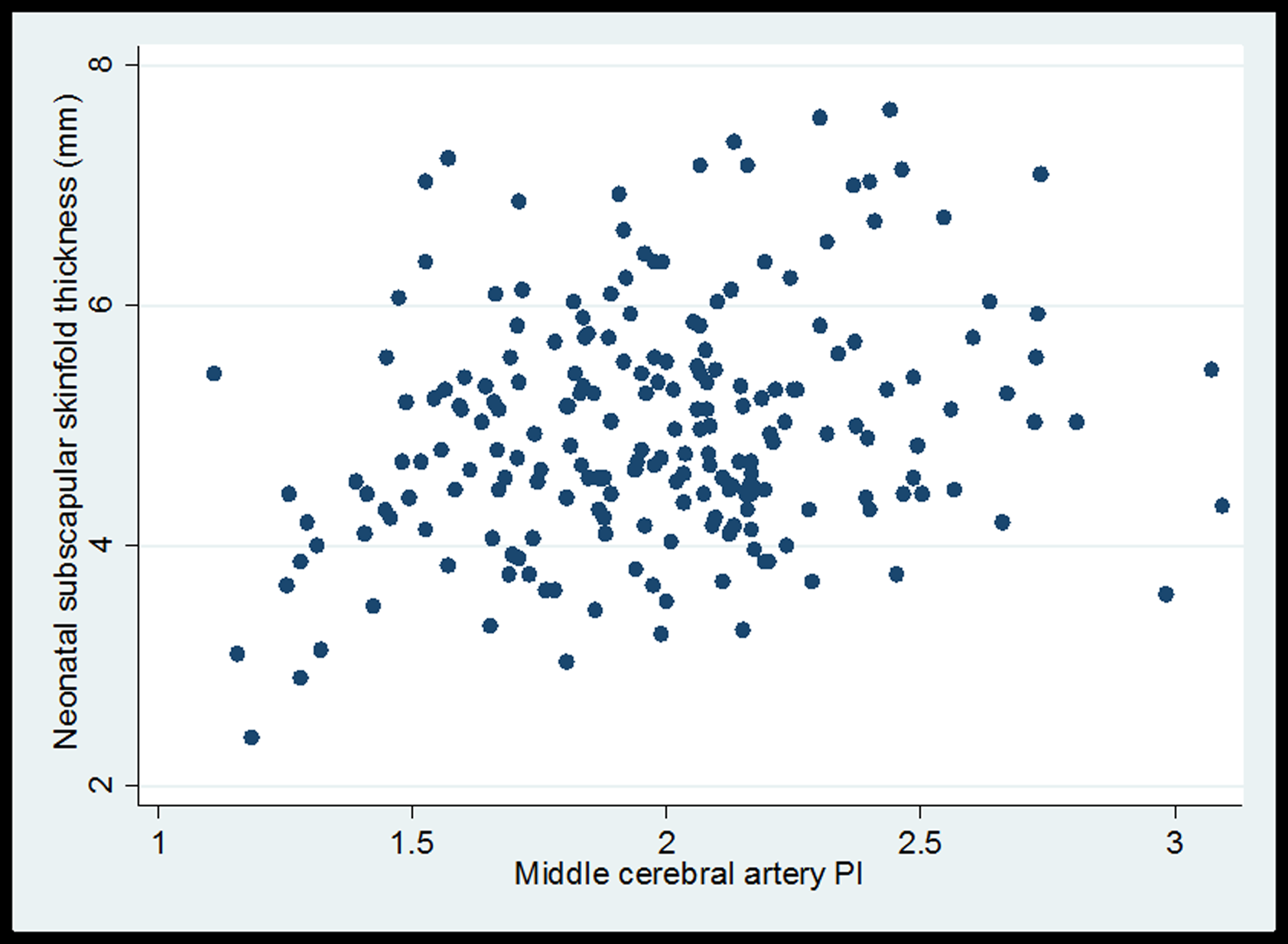

Supplement: Figure S3 — Scatterplot of neonatal subscapular skinfold thickness in relation to fetal middle cerebral artery pulsatility index (PI) in late gestation. (TIF) [file pone.0041759.s003.tif]

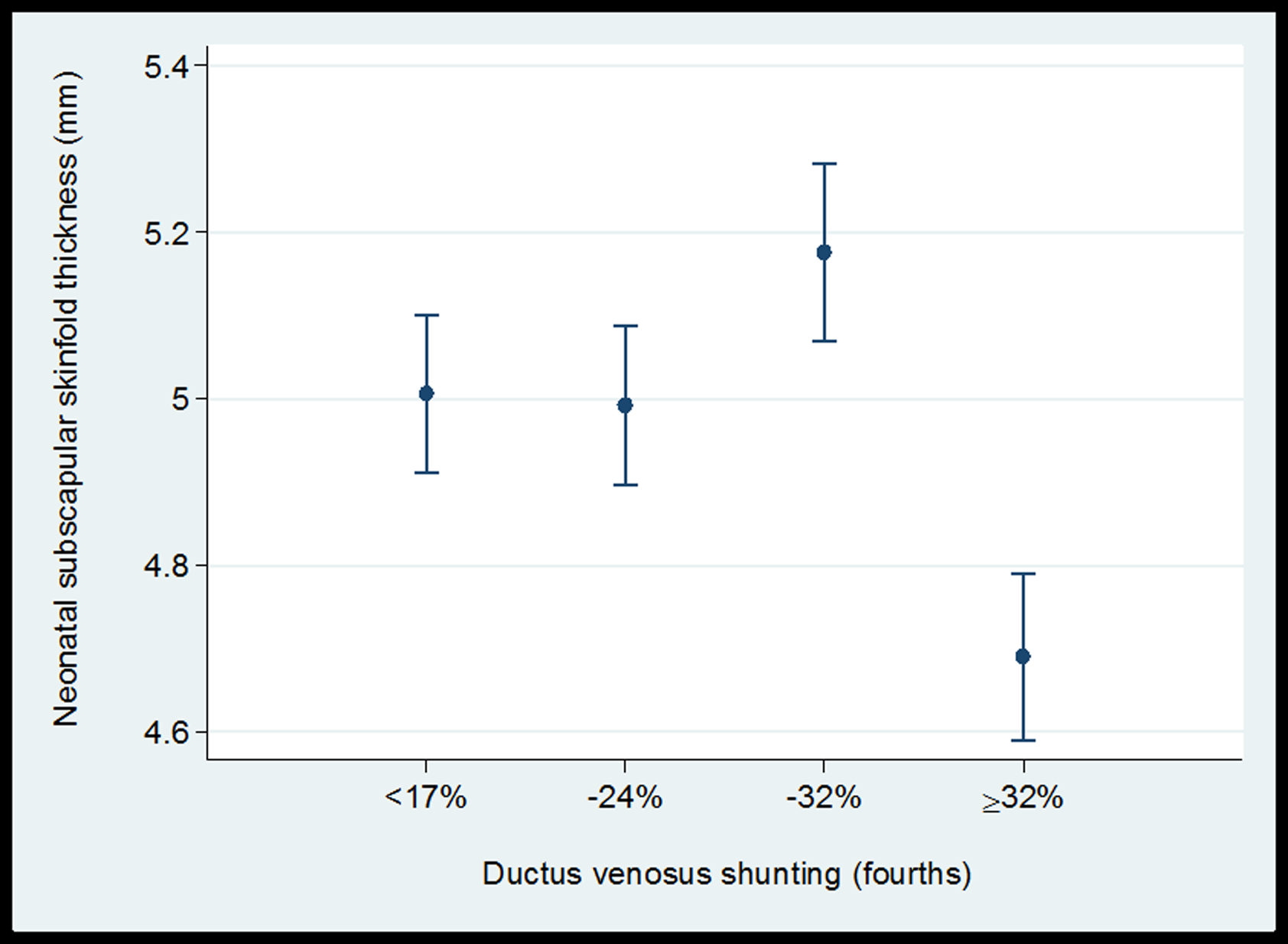

Supplement: Figure S4 — Neonatal subscapular skinfold thickness according to fourths of the distribution of ductus venosus shunting at 36 weeks gestation. Values are means and SEM. (TIF) [file pone.0041759.s004.tif]
